# A High-Pressure, High-Temperature Flow Reactor Simulating the Hadean Earth Environment, with Application to the Pressure Dependence of the Cleavage of Avocado Viroid Hammerhead Ribozyme

**DOI:** 10.3390/life12081224

**Published:** 2022-08-12

**Authors:** Kunio Kawamura, Mari Ogawa, Noriko Konagaya, Yoshimi Maruoka, Jean-François Lambert, Louis M. P. Ter-Ovanessian, Jacques Vergne, Guy Hervé, Marie-Christine Maurel

**Affiliations:** 1 Department of Human Environmental Studies, Hiroshima Shudo University, 1-1-1 Ozuka-higashi, Asaminami-ku, Hiroshima 731-3195, Japan; 2 Department of Primary Education, Yasuda Women’s University, 6-13-1 Yasuhigashi, Asaminami-ku, Hiroshima 731-0153, Japan; 3 Department of Nutritional Sciences, Yasuda Women’s University, 6-13-1 Yasuhigashi, Asaminami-ku, Hiroshima 731-0153, Japan; 4 Laboratoire de Réactivité de Surface—CNRS UMR 7197, Sorbonne Université—Campus Pierre et Marie Curie, 7 Quai Saint-Bernard, 75005 Paris, France; 5 Institut de Systématique, Evolution, Biodiversité (ISYEB), UMR 7205 CNRS MNHN UPMC EPHE, Muséum National d’Histoire Naturelle, Sorbonne Universités, CP. 50, 57 rue Cuvier, 75005 Paris, France; 6 Laboratoire BIOSIPE, Institut de Biologie Paris-Seine, Sorbonne Université, 7 Quai Saint-Bernard, 75005 Paris, France

**Keywords:** hammerhead ribozyme, Hadean Earth, RNA world, chemical evolution, high pressure, high temperature, research tool, kinetics

## Abstract

The RNA world hypothesis suggests that chemical networks consisting of functional RNA molecules could have constructed a primitive life-like system leading a first living system. The chemical evolution scenario of RNA molecules should be consistent with the Hadean Earth environment. We have demonstrated the importance of the environment at both high temperature and high pressure, using different types of hydrothermal flow reactor systems and high-pressure equipment. In the present study, we have attempted to develop an alternative easy-to-implement method for high-pressure measurements and demonstrate that the system is applicable as an efficient research tool for high-pressure experiments at pressures up to 30 MPa. We demonstrate the usefulness of the system by detecting the high-pressure influence for the self-cleavage of avocado hammerhead ribozyme (ASBVd(−):HHR) at 45–65 °C. A kinetic analysis of the high-pressure behavior of ASBVd(−):HHR shows that the ribozyme is active at 30 MPa and its activity is sensitive to pressures between 0.1–30 MPa. The surprising finding that such a short ribozyme is effective for self-cleavage at a high pressure suggests the importance of pressure as a factor for selection of adaptable RNA molecules towards an RNA-based life-like system in the Hadean Earth environment deep in the ocean.

## 1. Introduction

The RNA world hypothesis is well accepted for clarifying the early stage of chemical evolution and explains how simple chemical networks of biomolecules have evolved towards a life-like system [1,2]. Presumably, these steps from chaotic chemical networks to life-like systems were completed between 4.55–3.8 Ga since the oldest evidence of a living system can be seen at 3.5–3.8 Ga [3,4,5]. On the other hand, the scenario at the basis of the RNA world hypothesis should be consistent with the Hadean Earth environment before 3.8 Ga, which could have involved extreme conditions as compared to those after cell-type organisms emerged [6,7,8]. The temperature and pressure conditions of the aqueous phase are important since these physical factors primarily and sensitively determine the thermodynamics and kinetics of chemical reactivities. Knowledge about environments where the chemical evolution of RNA proceeded is being gradually clarified, and it appears that higher-temperature and higher-pressure environments than those of the present time have to be considered. Indeed, the environment for the chemical evolution of biomolecules on the Hadean Earth should have involved fairly extreme conditions as compared to the age after organisms appeared. At the beginning of the evaluation of the RNA world hypothesis under such extreme conditions, there were limited suitable research tools for analyzing chemical reactions occurring in such extreme conditions. Thus, we have developed and improved hydrothermal flow reactors (HFR) [7,9] and high-pressure reactors (HPR) [8,10] for evaluating the chemical behavior of biomolecules at high temperatures and high pressures. We demonstrated the usefulness of these research tools to explore the possibilities of chemical evolution under the simulated Hadean Earth environment. Both temperature and pressure as physical parameters are important for considering the functions of biomolecules [7,8,11,12].

In the presence of temperature-sensitive RNA motifs [13], ribozymes can control a gene in a temperature-dependent manner [14]. Moreover, some circular RNAs (such as viroids) are able to copy themselves in a host cell by a mechanism called “rolling circle”, where the initial sequence is recopied several times. Thus, ribozymes and viroids are considered as useful RNA models to investigate the chemical evolution before cell-type organisms. Recently, we showed that pressure and temperature influences on the avocado sunblotch viroid ASBVd compensate each other, on the basis of measurements of cleavage behaviors as a function of pressure and temperature using HPR [15]. The investigation suggested the influence of surrounding sequences of cleavage site.

At the same time, we noticed some points which would be improved for facilitating study of the chemical evolution of RNA molecules on the basis of our instrumentation studies. First, the reduction of sample volume and amount is very useful for this type of research, especially for experiments with valuable RNA molecules. Second, the extension of temperature limit and residence time in the high-pressure reactor would be useful for application to different types of chemical evolution conditions. Third, an easy-to-build system consisting of conventional materials is helpful to measure pressure-dependent reactions. Thus, we attempted to modify our hydrothermal flow reactor system to fit with high-pressure measurements since the hydrothermal flow reactor systems possess different mechanical characteristics from our HPR.

On the basis of the fundamental mechanics of the hydrothermal flow reactor system, we developed different types of hydrothermal flow reactor systems and demonstrated the usefulness of the methods in the field of the origins of life [7,16]. In addition, some successful improvements were applied to allow more practical applications, such as an in situ research tool system for solid-state catalysts and the continuous hydrothermal treatment reactor as an environmentally friendly technology [17]. On the other hand, our hydrothermal flow reactor system suffered a limitation, namely the relatively long periods of reaction monitoring; it was difficult for measurements of reaction behavior in the time scale from 10 min to several hours, since the HFR systems were originally developed for monitoring rapid reactions in the millisecond-to-second time scale. At the same time, there are limited suitable methods for our purposes [18]. The hydrothermal system can become more widely applicable for different reactions by improving this limitation.

In the present study, we first built a new type of high-pressure reactor system (HPR-30) and designed the protocol for high-pressure experiments on the basis of the techniques of HFR. Second, this system was applied for actual high-pressure measurements of the hammerhead ribozyme ASBVd(−):HHR designed from the minus strand of ASBVd. We attempted to compare the cleavage of ASBVd(−):HHR at pressures of 0.1 MPa (atmospheric pressure) and 30 MPa at 45–65 °C. Our results demonstrate that there is a specific pressure influence on ASBVd(−):HHR which is different from the influence observed on the original viroid. This study demonstrates that the newly developed high-pressure reactor is a useful and easy-to-build system for high pressure measurements.

## 2. Materials and Methods

### 2.1. High-Pressure Reactor (HPR-30)

**Figure 1:**
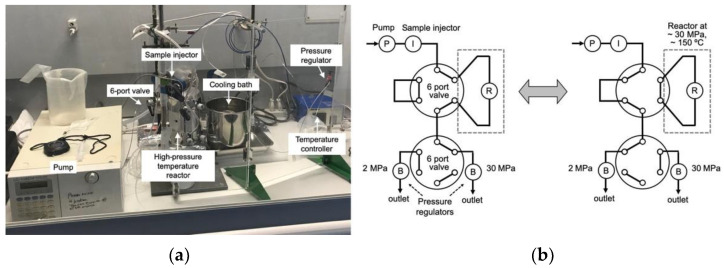
The high-pressure reaction system: photograph (**a**) and its valve system (**b**). By using the valve system to isolate the high-pressure tubing reactor, the high-pressure tubing is kept at high pressure for a long duration.

The Basic system of HPR-30 (Figure 1a,b) consists of a water reservoir, a high-pressure pump (LC-10ADvp, Shimadzu, Kyoto, Japan, or PU-4285, JASCO, Tokyo, Japan), a sample loop injector (VI-11, EYELA, Tokyo, Japan), a high-pressure/temperature reactor tubing coil (H-P/T coil) with heating aluminum blocks, a high-pressure switching valve (Rheodyne 7010, IDEX Health & Science, Oak Harbor, WA, USA), a cooling bath, two pressure regulators (PU-880, Upchurch Scientific, IDEX Health & Science, Oak Harbor, WA, USA), and a sampling port, designed based on our previous studies [7,9,16]. The high-pressure switching valve was used for changing the flow path to keep the tubing reactor at high pressure and high temperature in order to adjust the residence time by isolating the H-P/T coil from the main flow path. The flow scheme with switching valve is illustrated in Figure 1a,b. The switching 6-port valve is exposed to high temperature as well as the H-P/T-coil, which results in an upper temperature limit of 150 °C. The dead volume connecting the switching valve, which is also regarded as a part of H-P/T coil, was adjusted to less than 5% for the total volume of the H-P/T coil by shortening the connecting tubing lines. The pressure was controlled by the pressure regulator at values up to 30 ± 0.3 MPa. The timing for the switching valve after the sample injection and the sampling timing after the end of reaction were determined using 0.1 M HCl solution.

A 100 μL sample solution containing ASBVd(−):HHR was injected to introduce the sample into the H-P/T coil, where the sample was exposed at an adjusted pressure and temperature. The valve was then switched to isolate the sample solution from the main flow path to keep the sample solution in controlled pressure and temperature conditions (up to ~30 MPa and 150 °C). For the present ribozyme reactions, the pressure was adjusted to 30 MPa and the temperature between 45–65 °C. The high-pressure reaction was performed inside the tubing reactor isolated from the main flow. After the selected residence time period at high pressure and high temperature had elapsed, the valves were switched back to the sample solution to merge the main flow at pressure 2 MPa with the second back pressure regulator, and the sample was collected from the sampling port. The second back pressure regulator was used to reduce pulsating flow.

### 2.2. Materials

**Figure 2:**
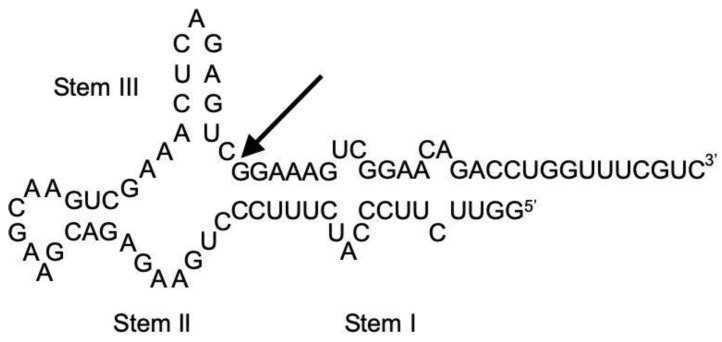
Sequence and secondary structure of ASBVd(−):HHR. The arrow indicates the self-cleavage site.

The ribozyme (whose sequence and secondary structure are shown in Figure 2) was prepared by in vitro transcription as described in our previous publication [19]. In addition, the same sequence of RNA was outsourced from FASMAC, Japan. We confirmed the purity of the RNA molecules by electrophoresis and HPLC, and there were no differences between the ribozymes prepared in our laboratory and those obtained from the company. All other reagents used for ribozyme reactions were of DNase- and RNase-free grade, and the reagents used for analysis were of analytical grade.

### 2.3. ASBVd(−):HHR Self-Cleavage at 0.1 MPa

An amount of 1.5 μL (0.132 μg) of ribozyme solution (8.8 μg ribozyme in 100 μL H_2_O) and 5.0 μL of 0.5 M HEPES solution were added to 0.2 mL PCR tubing. The tubing was settled in the thermal cycler (TP350, TaKaRa, Japan), where denaturation was performed at 94 °C for 1 min, followed by cooling down to 22 °C with a constant rate (3 °C/min) to form the ribozyme secondary structure prior to the ribozyme cleavage reaction. An amount of 5.0 μL of 0.5 M MgCl_2_ solution and 38.5 μL H_2_O were added to the PCR tubing and settled on the thermal cycler, which was adjusted at the target temperatures to run the cleavage reaction (total volume: 50 μL). To stop the cleavage reaction, 50 μL of 0.2 M EDTA solution at pH 8.0 was added. Samples were kept in a freezer at below −20 °C before the sample analysis.

### 2.4. ASBVd(−):HHR Self-Cleavage at 30 MPa

An amount of 15.0 μL (3.30 μg) of ribozyme solution (10 μg ribozyme/50 μL H_2_O) and 11.0 μL of 0.5 M HEPES solution were added to 0.2 mL PCR tubing. Denaturation was carried out using the same method as for the experiment at 0.1 MPa. An amount of 11.0 μL of 0.5 M MgCl_2_ solution and 73.15 μL H_2_O were added to the PCR tubing, with the total volume of mixture amounting to 110.0 μL. The mixture was injected through the sample injector to the H-P/T coil, the 600 μL sample was collected from the sampling port, and 40 μL of 0.5 M EDTA at pH 8.0 was added to stop the cleavage reaction. Samples were evaporated to ca. 50 μL using a centrifugation evaporator (CVE-2200, EYELA, Tokyo, Japan or Concentrator 5301, Eppendorf, Hamburg, Germany), kept in a freezer at below −20 °C before the sample analysis. The reaction conditions were the same as those at the atmospheric pressure except for using a higher concentration of ASBVd(−):HHR.

### 2.5. HPLC Analysis for the Products of ASBVd(−):HHR Self-Cleavage

ASBVd(−):HHR (79 nucleotide units in length) was cleaved into two components of 51 nt and 28 nt. The HPLC method was optimized for the separation of the ribozyme (79 nt) and its two cleavage components (28 nt and 51 nt). The separation behavior of RNA molecules will be discussed in detail in a work in preparation focused on HPLC behavior. HPLC analyses were carried out using an LC10A HPLC system (Shimadzu, Kyoto, Japan) or an LC-2040 Plus (Shimadzu, Kyoto, Japan) on an anion-exchange column (diameter 2 mm and length 75 mm, DNA-NPR, TOSOH, Tokyo, Japan) at a flow rate of 0.15 mL/min and using a gradient of 0.375–0.975 M NaCl at pH 9.0 with 7.5 M urea and 0.02 M 2-amino-2-hydroxymethyl-1,3-propanediol (Tris) buffer, or using an Infinity II (Agilent, Santa Clara, CA, USA) on an anion-exchange column (diameter 4.6 mm and length 75 mm, DNA-NPR, TOSOH, Tokyo, Japan) at a flow rate of 0.75 mL/min and using a gradient of 0.375–0.975 M NaCl at pH 9.0 with 7.5 M urea and 0.02 M Tris buffer. The HPLC method was modified on the basis of our previous method for oligonucleotide analysis [20] with the addition of a large amount of urea for denaturation of RNA [21,22]. All these HPLC analyses with the same gradient buffer, but using different systems with different sizes of the anion-exchange columns were consistent. The reaction sample volume was adjusted to 110–120 μL by the addition of H_2_O, and a 100 μL aliquot was injected into HPLC.

### 2.6. Secondary Structure Modeling of HHR:ASBVd(−)

To estimate the secondary structure of HHR:ASBVd(−), the RNAfold web server was used with different temperatures [23,24]. The parameters were set to default values except for changing the temperature. This estimation cannot account for pressure changes.

## 3. Results and Discussion

### 3.1. Construction of High-Pressure Reactor and Performance of HPR-30

The principle of HPR-30 is based on our previous HFR, which was originally designed for monitoring the millisecond-to-second time scale under hydrothermal conditions. The original HFR consists of a water reservoir, high-pressure pump, sample injector, high-temperature reactor with heating block, cooling bath, pressure controller, and sampling port. In the heating block, a narrow tubing reactor is settled for performing high-temperature reactions, and this was applied for the present HPR-30. The pressure inside the reactor tubing is controlled with the pressure regulator so as to be higher than the vapor pressure of water, in order to keep water in a liquid state. The residence time of the sample exposed to the high temperature is easily regulated by the flow rate of the pump or by using different sizes of tubing reactor. This enables short-time monitoring of the reactions, such as in the millisecond-to-second time scale (0.002–120 s) using different sizes of tubing (inner diameter: 0.015–0.25 mm). For instance, ca. 3–120 s residence times can be imposed using stainless-steel tubing of 0.25 mm inner-diameter and 2 m in length, which possesses ca. 0.1 mL inner volume, by regulating the flow rate to be within ca. 2 mL/min–0.05 mL/min. The system was initially built for monitoring short time scale reactions so that the residence time is suitable for hydrothermal reactions with relatively fast reaction rates. However, this short time scale is not well suited to the reaction time scale of ASBVd(−):HHR, which generally proceeds within the time scale from a few minutes to a few hours [19]. It is necessary to be able to work under different conditions for the measurement of ribozyme reactions.

Initially, we attempted to adjust the flow rate to a very low value. The longest residence time exposed at high pressure is limited to 100 min, corresponding to the minimum adjustable flow rate of 0.001 mL/min. However, controlling the flow rate at 0.001 mL/min is not practical because pressure regulation is difficult and the accuracy of flow rate control decreases at a very low flow rate. Thus, we adopted the method mentioned in the experimental section, which is applicable in principle to much longer reaction times at high pressures than those tested in this study, of a few hours and longer (Figure 1). The flow path to the high-pressure tubing is switched to isolate the high-pressure tubing from the main flow of the system, as shown in the left of Figure 1b, which enables the high-pressure tubing to be a high-pressure vessel which is maintained for the long reaction time scale. There is no upper limit on the residence time unless the sample leaks from the flow reactor tubing.

The reaction pressure in HFR, for instance at 250 °C, is normally controlled at 5–10 MPa since the vapor pressure of water at 250 °C is ca. 4 MPa. We have previously determined that this kind of system is resistant up to 400 °C with a pressure normally adjusted to below 30 MPa. According to our previous studies regarding the hydrolytic degradations of simpler biomolecules, we did not observe any pressure influence under hydrothermal conditions in such cases [7,9,16]. It is reasonable to think that the conformation of simple molecules in the activated state is less dependent on pressure. In the present study, the performance of the system needed to be evaluated with a reaction system using complex organic molecules, such as ribozyme cleavage, for which the pressure influence has already been identified in previous studies [8,10,15].

The maximum pressure which can be imposed is normally determined by the limit pressure of the system components. In the present system, these components consist of the pump, the sample injector, the valve, the pressure regulator, etc. Thus, we built a system which can be used at up to 30 MPa by considering the upper-limit pressure of the pump. In other words, this fact indicates that the maximum pressure could be increased by using components which can be used at higher pressures as a future improvement.

The timing at which the sample solutions reach the inlet and the outlet of the high-pressure tubing was determined by injecting 0.1 M HCl solution and checking the color change of pH test paper. The timing for recovering the samples exposed to high pressure after the switching valve was changed to the main flow was also determined in the same way. In addition, we confirmed that we can recover the RNA molecules by switching the 6-port valves properly. According to our previous study regarding the diffusion of sample solutions, it was estimated that the sample solution is diluted by a factor of 1.55 [25] in our typical hydrothermal flow reactors. From the measurement of the elution of HCl solution in the present study, the sample is approximately diluted by a factor of 2 at a flow rate of 0.2 mL/min. This indicates that the 0.1 mL sample solution should be recovered in within 1 min. However, we confirmed that the recovery of 0.4–0.6 mL samples gives better results for the ASBVd(−):HHR cleavage reaction. This seems to be due to the low concentration of the ASBVd(−):HHR, resulting in stronger dilution as compared to the 0.1 M HCl solution. Thus, we adjusted the recovery of 0.4–0.6 mL samples at the sampling port for the 0.1 mL injection of ASBVd(−):HHR solution. Furthermore, for the sample analysis by HPLC, we found that the concentration was occasionally useful for sample analysis by using a centrifugation evaporator to adapt the sample volume for HPLC analysis. The concentration did not affect the cleavage reactions provided that the reaction was stopped by the addition of EDTA.

Based on the reaction profiles using HPR-30, we confirmed that the HPR-30 showed good performance for monitoring reactions at high pressures at temperatures up to 65 °C and a residence time of 120 min at 30 MPa. Theoretically, the residence time of the sample exposed to high pressure and high temperature is to be controlled for a very long time as compared to our previous methods [7,9,16] and literature reports using flow systems [18]. On the other hand, the upper temperature limit should be 150 °C since that is the limit for the switching 6-port valves. The accuracy of the reaction curves in the next section indicates that an influence of the dead volume connecting between the high-pressure tubing and the switching 6-port valves was not observed.

### 3.2. Cleavage of ASBVd(−):HHR at 0.1 MPa and 30 MPa at 45–70 °C

**Figure 3:**
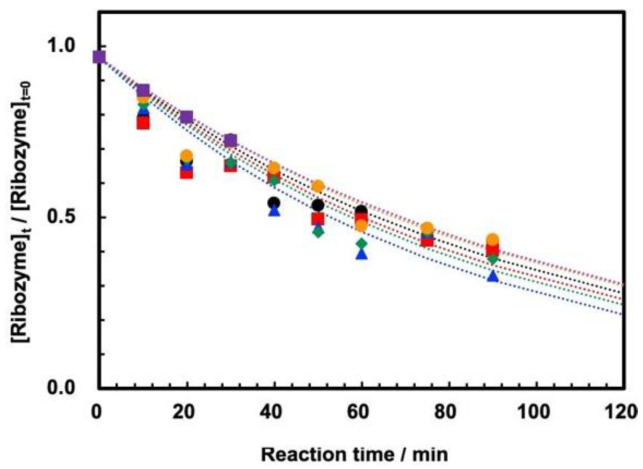
Reaction curves for self-cleavage of ASBVd(−):HHR at 0.1 MPa at 45–70 °C. [ASBVd(−):HHR] = 0.132 μg/50 μL solution. [HEPES] = 0.05 M, [MgCl_2_] = 0.05 M, pH = 8.0. Black circles: 45 °C, red squares: 50 °C, blue triangles: 55 °C, green diamonds: 60 °C, orange circles: 65 °C, purple squares: 70 °C.

The cleavage of ASBVd(−):HHR using PCR test tubing was carried out at temperatures of 45–70 °C at 0.1 MPa. Reaction curves are shown in Figure 3. According to our previous study of ASBVd(−):HHR, the self-cleavage of ribozyme proceeds to form two oligonucleotides with 28 and 51 nucleotide units (28 nt and 51 nt). The self-cleavage reaction obeys first-order kinetics, so the lower concentration should basically not affect the reaction behavior (Supplementary data, Figure S1). The HPLC conditions were optimized for separation of ASBVd(−):HHR (79nt) and its two cleavage products (51nt, 28nt) by using a gradient mode for HPLC using NaCl in the presence of 7.5 M Urea. Three main peaks were observed, one of which corresponds to the ASBVd(−):HHR, with a retention time of ~20 min on the column with 4.6 mm diameter. A treatment for denaturing of ASBVd(−):HHR prior to HPLC analysis did not affect the retention time of ASBVd(−):HHR. According to the literature [21,22], running HPLC at high temperatures seems to be useful. However, this is not the case for the present system, so it was possible to run HPLC at 35 °C. The retention time of one of the two reaction products (~12 min) was confirmed as being identical to that of the oligonucleotide with 28 nucleotide units, so it is reasonable that the other peak at ~18 min corresponds to the 51 nt fragmentation product. Thus, we are able to follow the self-cleavage reaction straightforwardly by using the present HPLC system.

The measurements using either the ribozyme prepared by in vitro transcription in our laboratory or that prepared commercially in FASMAC did not show any difference in their cleavage behavior. According to our previous study, the self-cleavage is dominant at temperatures up to 60 °C, while partially random degradative hydrolysis of phosphodiester bonds within the ribozyme sequence occurs with increasing temperature, especially over 65 °C [19]. We confirmed that the reaction was observable up to 70 °C in batch reactions, that it still followed first-order kinetics, and that there was no notable influence of the random degradation of the ribozyme on the cleavage analysis. The first-order rate constants at temperatures of 45–70 °C are summarized in Table 1. The influence of random hydrolysis is not notable, so the determination of the first-order rate constants was based on the ratio of 79 nt among the total amount of 28 nt, 51 nt, and 79 nt as per our previous study [19]. The temperature dependence of the rate constants shows that they are maximum at 55 °C. This is consistent with our previous study [19]. In addition, this is reasonable since the cleavage rate of ASBVd was also maximum at 55 °C [15].

**Figure 4:**
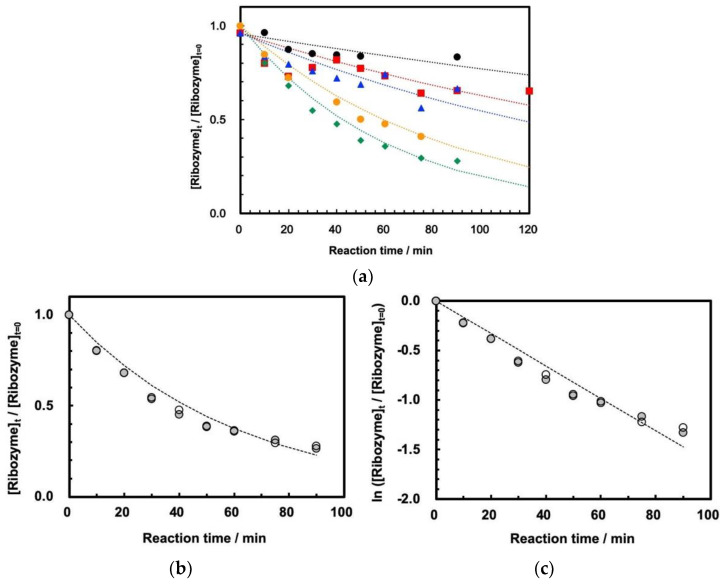
Reaction curves for self-cleavage of ASBVd(−):HHR at 30.0 MPa at 45–65 °C. [ASBVd(−):HHR] = 3.30 μg/110 μL solution. [HEPES] = 0.05 M, [MgCl_2_] = 0.05 M, pH = 8.0. (**a**) Reaction curves at 45–65 °C. Black circles: 45 °C, red squares: 50 °C, blue triangles: 55 °C, green diamonds: 60 °C, orange circles: 65 °C. (**b**) Duplicated measurements of reaction curves at 60 °C. (**c**) First-order rate plots for the duplicated measurements of reaction curves at 60 °C.

On the other hand, the measurements of self-cleavage of ASBVd(−):HHR were successfully measured for the first time at 30 MPa at temperatures of 45–65 °C by using HPR-30. The reaction curves at temperatures of 45–65 °C are shown in Figure 4a. We confirmed that the reaction profile is well reproducible by duplicating the experiments at 60 °C (Figure 4b). This indicates that ribozyme cleavage proceeded well inside the tubing reactor at 30 MPa at 45–65 °C, that the reaction was stopped completely by the addition of EDTA solution, and that evaporation of the sample mixture did not affect the cleavage reaction. The first-order kinetic plots show that the reaction basically follows first-order kinetics at 30 MPa (Figure 4c), although the plots seem to diverge from the estimated first-order kinetics curve at longer reaction times. The first-order rate plots at 30 MPa at 45–65 °C are summarized in Figure S2 (Supplementary Materials), and the rate constants are shown in Table 1 for comparison with the rate constants at 0.1 MPa.

The present measurements using HPR-30 demonstrate that the HPR-30 system allows us to follow the ribozyme behavior at 30 MPa using a small amount of ribozyme (ca. 3.0 μg) and a small volume of sample solution (100 μL in sample loop volume). The measurements at high pressure for relatively long residence time up to 120 min were also successful, without leaking of the liquid from the lines of the system at 30 MPa. Furthermore, the present system is relatively simple to build, so that high-pressure measurements would be easily applicable in different fields of chemical and biochemical processes.

### 3.3. Influence of High Pressure on the Cleavage Behavior of ASBVd(−):HHR

Although there are some extensive studies regarding the enzymatic activities of protein enzymes at high pressures [10,11,26,27,28,29], high-pressure studies on ribozymes are scarce [8,10,15]. Here, we confirmed that the cleavage reaction forming two components of 28 nt and 51 nt RNAs proceeded at both 0.1 and 30 MPa at 45–65 °C. This is consistent with our previous study, where ASBVd(−):HHR showed that the cleavage reaction proceeds in the temperature range of 10–70 °C [15]. The reaction rate constants are relatively small as compared to other hammerhead ribozymes [30,31]. It was observed that random hydrolysis of the ribozyme proceeds in parallel with the cleavage reaction, and that the ratio of random hydrolysis to cleavage increases at temperatures over 65 °C. This is also consistent with our previous study [19]. Although minor products other than 28 nt and 51 nt RNAs were observed by HPLC analysis corresponding to random hydrolysis, the cleavage reaction was dominant under our pressure and temperature conditions.

**Figure 5:**
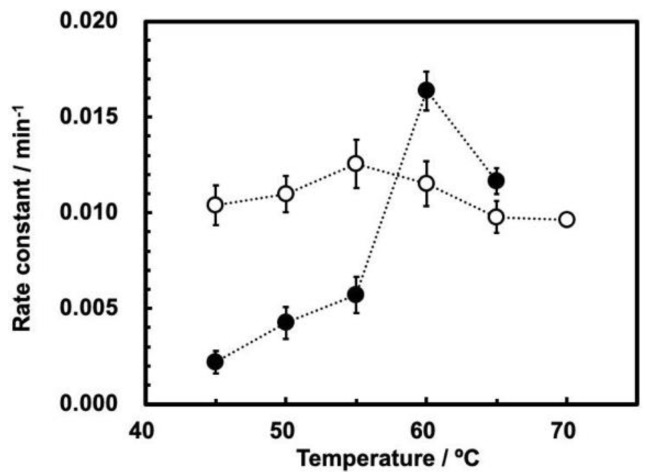
Rate constants of self-cleavage of ASBVd(−):HHR at 0.1 MPa (open circles) and 30.0 MPa (closed circles) as a function of temperature. Reaction conditions are the same as those shown in Figure 3 and Figure 4.

The profiles of the rate constants vs. temperature at 0.1 and 30 MPa (Figure 5) suggest the following comments.

First, the rate constants of ribozyme cleavage are significantly smaller at 30 MPa than at 0.1 MPa at temperatures up to 55 °C; ribozyme cleavage is inhibited by a high pressure up to 55 °C. Second, the maximum rate constant was observed at 55 °C at 0.1 MPa, and at 60 °C at 30 MPa. This difference suggests that the cleavage of the ribozyme basically follows the same reaction mechanism to the original ASBVd [15], while the maximum temperatures are somewhat different between 0.1 MPa and 30 MPa. Third, the influence of temperature at 0.1 MPa is moderate, as the ratio between rate constants at 55 °C and 45 °C is ca. 1.25 times for ASBVd(−):HHR. This is similar to the ratio between rate constants at 55 °C and 45 °C for ASBVd at the same pressure [15]. In contrast, the rate constant at 30 MPa is more sensitive to temperature, as the rate constant at 60 °C is 12 times higher than that at 45 °C. The cleavage reaction rates at 30 MPa is actually higher than those at 0.1 MPa at 60–65 °C. This seems to be at odds with the results published in reference [15]; as can be seen from Figure 3a, which shows the effect of temperature on the reaction rates at different pressures, the rates consistently decrease with increasing pressure, and they never become greater (at 65 °C, the rate at 25 MPa is smaller than at 0.1 MPa). Furthermore, the temperature dependence for ASBVd(−) at higher pressures up to 200 MPa was moderate in comparison to the ribozyme [15]. The ratio between the rate constants at 55 °C and at 45 °C is less than a factor of 3 at all pressures between 0.1–200 MPa.

In addition, as mentioned above, the cleavage reaction does not seem to perfectly fit first-order kinetics in the present study (Figure 4c). It is known that the cleavage of a hammerhead ribozyme from Chrysanthemum chlorotic mottle viroid consists of a two-component reaction mechanism, including fast and slow reactions [10]. According to our previous investigation, these are dependent on the interactions of three-dimensional structures due to surrounding sequences of the active center of the ribozyme part of the viroid [15]. This is also consistent with the assumption that the associate formation prior to the self-cleavage becomes weak with increasing temperature [32]. Thus, we assumed that the complex kinetics are due to conformational effects of those three-dimensional structures. Thus, the temperature- and pressure-related behaviors of the cleavage of ASBVd(−):HHR in the present study would suggest that the difference between 0.1 and 30 MPa is due to different conformational change of the ribozyme at 60 °C, where the absence of surrounding long sequences in the ribozyme weakens the core structure of the catalytic center at 30 MPa.

**Figure 6:**
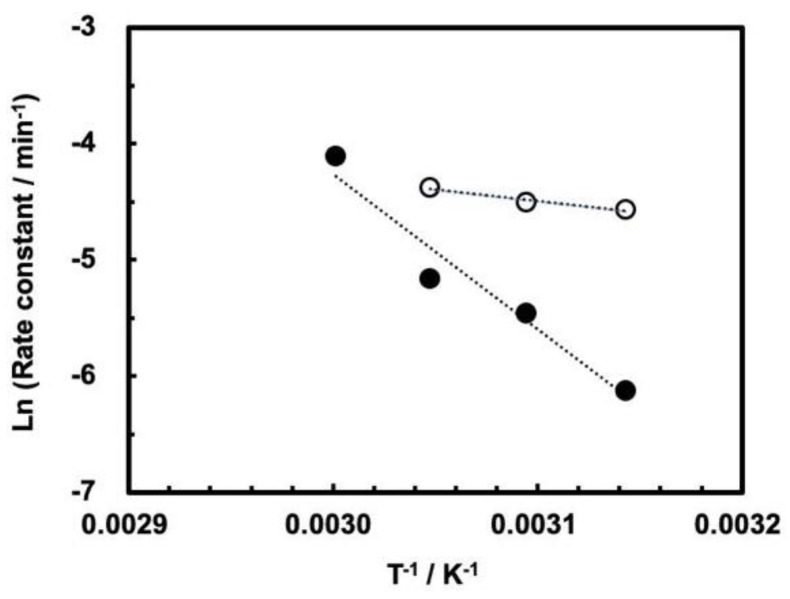
Arrhenius plots for self-cleavage of ASBVd(−):HHR at 0.1 MPa (open circles) and 30.0 MPa (closed circles). Reaction conditions are the same as those shown in Figure 3 and Figure 4.

The cleavage reaction obeys the Arrhenius plots for the cleavage rate constants up to the maximum rate, that is, at 55 °C for 0.1 MPa and at 60 °C for 30 MPa, as shown in Figure 6. The apparent activation energies in the temperature ranges up to the maximum rate have been calculated (Table 2). Although we do not fully understand the origin of the trends shown in Figure 5, we assume the following kinetic reasons on the basis of the apparent activation energies. First, the difference between 0.1 MPa and 30 MPa is large as compared to the case of ASBVd(−) viroid, where the behaviors of temperature dependence seems to be parallel for ASBVd(−) viroid at pressures of 0.1–200 MPa. Second, the fact that the activation energy at 30 MPa is fairly high suggests that the enzymatic function for self-cleavage at a specific position is less active at 30 MPa, so the activation energy is not efficiently reduced by the ribozyme. However, the presence of an optimum temperature indicates that the enzymatic function of the ribozyme is partially active at 30 MPa. In addition, this fact is consistent with our previous results showing that self-cleavage is affected by surrounding sequence of the ASBVd(−) viroid [15,33]. At the same time, we need to assume that a specific three-dimensional structure is partially maintained in order to ensure cleavage at the particular position -CG- between the 51st and 52nd nucleotide residues within the ribozyme.

**Figure 7:**
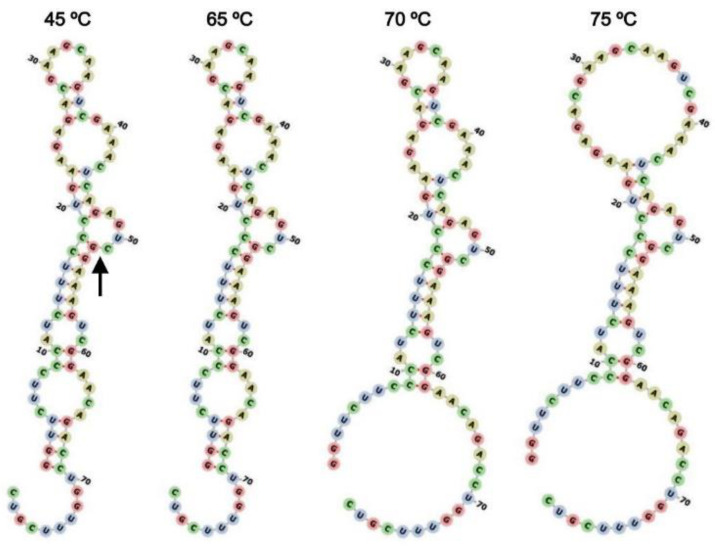
Estimated secondary structures of ASBVd(−):HHR as a function of temperature. The arrow indicates the self-cleavage site.

Modeling of secondary structures of the ribozyme (Figure 7) indicates that the centroid secondary structure of ASBVd(−):HHR starts to change between 65 °C and 70 °C, but the estimation does not account for the pressure influence. In spite of this shortcoming, this is consistent with the optimum reaction rate observed at 55–60 °C for ASBVd(−):HHR at 0.1 and 30 MPa. Although the partial disappearance of the hydrogen bonding within the secondary structure gradually starts at 70–75 °C. These observations would reflect the fact that the cleavage of the ribozyme at 65 °C and 70 °C proceeded dominantly rather than the random hydrolysis of the ASBVd(−):HHR, where the ribozyme self-cleavage is still active at 65 and 70 °C.

Comparison of the rate constants at 0.1 MPa and 30 MPa demonstrates that the reaction rate decreases with increasing pressure at 55 °C and below, and the reaction rate increases with increasing pressure at 60 °C and 65 °C. The activation volume is positive at 55 °C and below and negative at 60 and 65 °C. The positive activation volume suggests that there is desolvation of water molecules at the activated complex, but the negative activation volume suggests that there is solvation by water molecules within the activated complex. The activation volume can be estimated from the data at 0.1 MP and 30 MPa, although the accuracy of calculation is poor due to only using two different pressures. The roughly estimated values of ΔV^‡^ are 141 mL/mol at 45 °C, 87 mL/mol at 50 °C, 61 mL/min at 55 °C, −33 mL/mol at 60 °C, and −17 mL/mol at 65 °C. These values may suggest that a number of water molecules of one or higher contributes to the self-cleavage reaction since the mol volume of water is 18 mL/mol. The increase of the temperature to 60 °C would cause a change of conformation favoring the fixation of water molecules during the formation of the transition state, thus leading to a decrease of the reaction volume, down to negative values; this explains why, at high temperatures, there is an increase of the rate when the pressure increases. We are currently designing a high-pressure flow reactor adapted to run the reactions at pressures higher that 30 MPa so that a detailed analysis of the pressure dependence for ASBVd(−):HHR and inspection of hydration and dehydration [34] can be carried out.

A pressure of 30 MPa appears frequently in submarine vent systems deep in the ocean, corresponding to 3000 m in depth. In addition, the length of ASBVd(−):HHR is moderate as compared to functional RNA in nature and in synthetic RNA by the in vitro selection technique [35]. Thus, the fact that the cleavage reaction rate decreases with increasing pressure at temperatures up to 55 °C is useful for considering the compensation effect between pressure and temperature for primitive functional RNA molecules. In addition, the fact that the self-cleavage of ASBVd(−):HHR is fairly sensitive to pressures between 0.1 and 30 MPa suggests that pressure is an important factor for the selection and chemical evolution of RNA molecules in an RNA world compatible with the Hadean Earth environment. Finally, these data prove that our new high-pressure research tool would be very useful for investigating chemical evolution at high pressures at various temperatures since the system can be readily constructed from commercially available equipment and materials, as well as being suitable for a small volume of samples.

## 4. Conclusions

We have demonstrated a new type of flow reactor system for running high-pressure reactions, which enables us to carry out the cleavage reaction of ASBVd(−):HHR at 30 MPa with small amounts of RNA. By using the present system, we observed the temperature dependence of ASBVd(−):HHR at 45–65 °C at 30 MPa. The comparison of the reaction behaviors at 0.1 MPa and 30 MPa and different temperatures implies that the efficiency for the self-cleavage of the ribozyme is not very high at 30 MPa as compared to that at 0.1 MPa below 60 °C. The comparison between ASBVd(−):HHR and ASBVd(−) showed that the surrounding sequences of ASBVd(−) are very effective for preserving the core structure of the ribozyme. Our high-pressure reactor system can be constructed simply from commercially available equipment and materials (except for heating blocks), resulting in effective studies on the chemical evolution of RNA and other biomolecules under high pressures relevant for chemical evolution. The present method would be a powerful research tool for the investigation and exploration of the co-chemical evolution between RNA and peptides on Hadean Earth [36] as well as the possible origin of life for an exoplanet.

## Figures and Tables

**Table 1:** Rate constants (min^−1^) for the cleavage reaction of ASBVd(−):HHR at 0.1 and 30 MPa.

Temperature (°C)	First-Order Rate Constant (min^−1^)	
	0.1 MPa ^1^	30 MPa ^2^
45	(1.04 ± 0.07) × 10^−2^	(2.18 ± 0.58) × 10^−3^
50	(1.10 ± 0.09) × 10^−2^	(4.25 ± 0.84) × 10^−3^
55	(1.25 ± 0.13) × 10^−2^	(5.69 ± 0.94) × 10^−3^
60	(1.15 ± 0.12) × 10^−2^	(1.64 ± 0.10) × 10^−2^
65	(9.78 ± 0.84) × 10^−3^	(1.17 ± 0.07) × 10^−2^
70	(9.62 ± 0.23) × 10^−3^	NA

^1^ Batch reaction: [ASBVd(−):HHR] = 0.132 μg/50 μL solution. [HEPES] = 0.05 M, [MgCl_2_] = 0.05 M, pH = 8.0. ^2^ Flow reaction: [ASBVd(−):HHR] = 3.30 μg/110 μL solution. [HEPES] = 0.05 M, [MgCl_2_] = 0.05 M, pH = 8.0.

**Table 2:** Apparent activation energy (E_a_) for the self-cleavage of ASBVd(−):HHR at 0.1 and 30 MPa.

Pressure (MPa)	E_a_ (kJ mol^−1^)
0.1	16.3 ± 4.0
30	111.5 ± 18.5

## Data Availability

Not applicable.

## Supplementary Materials

The following supporting information can be downloaded at https://www.mdpi.com/article/10.3390/life12081224/s1. Figure S1: First-order rate plots for the self-cleavage of ASBVd(−):HHR at 0.1 MPa; Figure S2: First-order rate plots for the self-cleavage of ASBVd(−):HHR at 30 MPa.
