# Supplementary material for: A High-Pressure, High-Temperature Flow Reactor Simulating the Hadean Earth Environment, with Application to the Pressure Dependence of the Cleavage of Avocado Viroid Hammerhead Ribozyme
Source: Life (Basel). 2022 Aug 12;12(8):1224. doi: 10.3390/life12081224 (PMC9410335; doi:10.3390/life12081224)
Supplement: Supplementary file 1 [file life-12-01224-s001.zip › life-1853731-supplementary.pdf]

Supplementary data

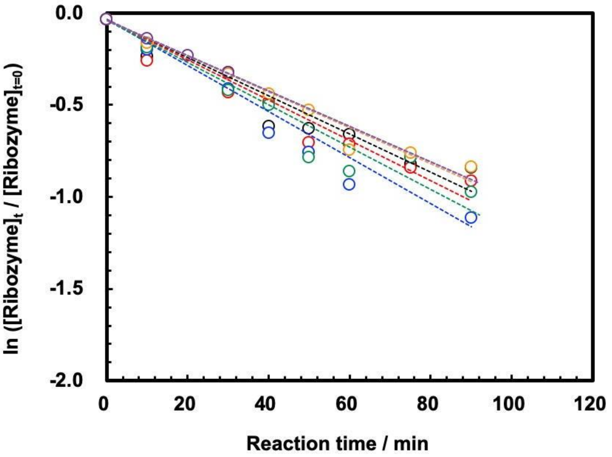

Figure S1. First-order rate plots for the cleavage of ASBVd(-):HHR at 0.1 MPa. [ASBVd(-):HHR] = 0.132 μg / 50 μL solution. [HEPES] = 0.05 M, [$MgCl_2$] = 0.05 M, pH = 8.0. Black circles: 45 °C, red circles: 50 °C, blue circles: 55 °C, green circles: 60 °C, orange circles: 65 °C, purple circles: 70°C.

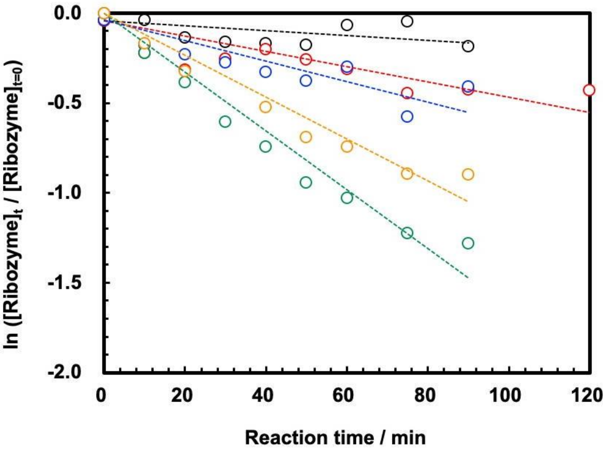

Figure S2. First-order rate plots for the cleavage of ASBVd(-):HHR at 30.0 MPa. [ASBVd(-):HHR] = 3.30 μg / 110 μL solution. [HEPES] = 0.05 M, [$MgCl_2$] = 0.05 M, pH = 8.0. Black circles: 45 °C, red circles: 50 °C, blue circles: 55 °C, green circles: 60 °C, orange circles: 65 °C.
